# Aldosterone synthase inhibitors for hypertension: A breakthrough facing barriers to adoption

**DOI:** 10.1371/journal.pmed.1004900

**Published:** 2026-02-09

**Authors:** Luke J. Laffin, Steven E. Nissen

**Affiliations:** 1 Section of Preventive Cardiology and Rehabilitation, Department of Cardiovascular Medicine, Cleveland Clinic Foundation, Cleveland, Ohio, United States of America; 2 Cleveland Clinic Coordinating Center for Clinical Research, Cleveland Clinic Foundation, Cleveland, Ohio, United States of America

## Abstract

In this Perspective, Luke Laffin and Steven Nissen discuss the promise and challenges of aldosterone synthase inhibitors as the next breakthrough in the treatment of uncontrolled hypertension, the number one risk factor for cardiovascular disease.

## Teaser

Despite many antihypertensive options, uncontrolled hypertension remains pervasive. Aldosterone synthase inhibitors show robust blood pressure reductions and have the potential to reshape hypertension treatment, though cost and access may impact broad adoption.

**Fig 1:**
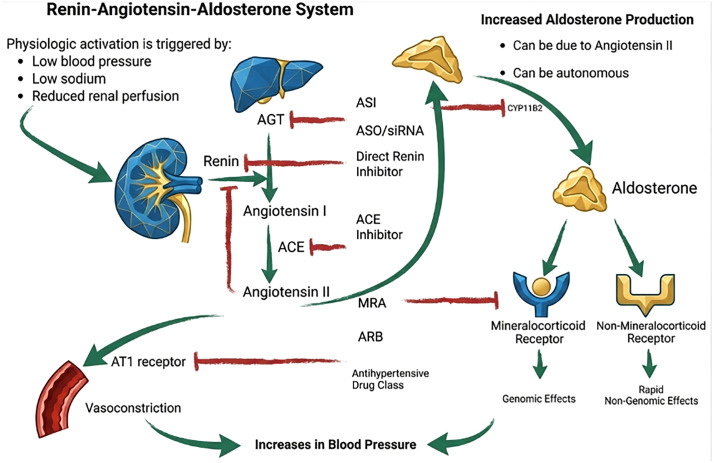
Site of action for common antihypertensive drug classes targeting the Renin-Angiotensinogen-Aldosterone pathway. Physiological activation of the renin-angiotensin-aldosterone system (RAAS) occurs with reduced renal perfusion, low blood pressure, or low sodium. Angiotensinogen is constitutively produced, predominantly by the liver, and circulates at concentrations that exceed the catalytic requirements of renin, rendering renin release the principal rate-limiting step of the RAAS. Aldosterone is synthesized in the adrenal cortex, where angiotensin II and potassium regulate aldosterone synthase expression and aldosterone production. Autonomous aldosterone production arises from intrinsic adrenal zona glomerulosa activity, frequently due to somatic mutations, resulting in aldosterone secretion that escapes normal RAAS feedback control. ACE, angiotensin converting enzyme; AGT, angiotensinogen; ARB, angiotensin receptor blocker; ASI, aldosterone synthase inhibitor; ASO, antisense oligonucleotide; AT1, angiotensin II Type 1; MRA, mineralocorticoid receptor antagonist; SiRNA, small-interfering RNA.

The treatment of hypertension worldwide is dominated by generic drugs with at least 10 classes of antihypertensive medications widely available to prescribers and patients. Three drug classes are recommended as initial pharmacotherapy and include a thiazide-type diuretic, dihydropyridine calcium channel blocker, and either an angiotensin converting enzyme inhibitor or angiotensin receptor blocker (see Fig 1). With blood pressure (BP) measurements greater than 140/90 mm Hg, single-pill combination therapy, including at least two of the above classes, is advised. Resistant hypertension is defined as uncontrolled hypertension while taking three BP-lowering medications. Guidelines recommend the use of mineralocorticoid receptor antagonists (MRAs) among patients with resistant hypertension, with recent evidence also supporting the use of amiloride in these patients [1,2]. Spironolactone, the most well-studied MRA, effectively lowers BP, but remains under-prescribed for resistant hypertension largely because of clinician concerns about hyperkalemia, coupled with fears of antiandrogenic side effects [3].

Even with a multitude of available drugs and drug classes, hypertension remains the number one modifiable risk factor for cardiovascular disease worldwide [4]. Reasons for the apparent disconnect between the wide availability of effective pharmacotherapy, yet the persistently high prevalence of uncontrolled hypertension are multifactorial, including prescriber inertia, medication side effects, and patient lifestyle factors such as obesity and excess sodium consumption.

It remains uncertain how best to stem the tide of uncontrolled hypertension and its associated morbidity and mortality; however, renewed attention to mechanisms underlying the pathogenesis of hypertension is required. Key developments have emerged in the past 5 years that highlight renewed attention to one such mechanism—aldosterone dysregulation. This includes recognition that up to 30% of patients with resistant hypertension have primary aldosteronism, an endocrine disorder where the adrenal glands overproduce aldosterone [5]. Further, beyond overt primary aldosteronism, a spectrum of aldosterone dysregulation (sometimes referred to as autonomous aldosterone production) exists among patients with primary hypertension, resulting in BP elevations and contributing to excess cardiovascular risk [6]. Recently, highly selective aldosterone synthase inhibitors (ASIs) have entered development and reduce aldosterone biosynthesis, as well as BP, safely and effectively. They also may allay clinician concerns about the side effects attributable to MRAs, particularly spironolactone. The following provides an overview of the data supporting the use of ASIs for the treatment of hypertension, while also providing perspective about how ASIs may be used when approved by global regulatory agencies.

## Safe and effective blood pressure lowering by ASIs

The current generation of highly selective ASIs includes four drugs: baxdrostat, lorundrostat, dexfadrostat, and vicadrostat. Among these, baxdrostat and lorundrostat are the most advanced in development and are actively being investigated for treatment of hypertension. Dexfadrostat was studied among patients with primary aldosteronism, and vicadrostat is being developed as a combination product with empagliflozin for the treatment of chronic kidney disease and heart failure.

Recent clinical results with ASIs have established that targeting aldosterone production produces BP reductions that are both substantial and consistent across populations with uncontrolled or resistant hypertension, while avoiding many of the liabilities that have historically limited mineralocorticoid receptor antagonism, including antiandrogenic or progestogenic side effects. Across programs, the magnitude of placebo-adjusted systolic BP lowering, between 7 and 12 mm Hg, places ASIs squarely in the range of the most effective antihypertensive drug classes currently in use [7–9]. Importantly, this efficacy was achieved without suppression of cortisol synthesis (which was seen when an earlier generation ASI, osilodrostat, was evaluated for the treatment of hypertension) and with relatively low rates of hyperkalemia and hyponatremia [10]. By suppressing aldosterone synthesis itself, ASIs may also uniquely address both non-genomic aldosterone signaling and the paradoxical return of aldosterone secretion during chronic renin–angiotensin system inhibition (known as aldosterone escape) (Fig 1). These are pathways that likely contribute to persistent BP elevation despite otherwise guideline-concordant therapy. Overall, ASIs represent a high-potency mechanism acting on a dominant and underappreciated driver of BP elevation.

All late-stage ASI trials to date have evaluated these agents on top of existing antihypertensive regimens, sometimes after protocol-mandated optimization of background therapy. This design choice has practical advantages but may blunt or obscure the full physiological impact of aldosterone suppression, potentially underestimating the standalone contribution of ASIs. These data challenge the notion that resistant hypertension is simply a failure of adherence or drug selection; rather, they reinforce the concept that aldosterone biology frequently remains inadequately addressed despite multidrug therapy. Seen through this lens, the success of ASIs is not surprising and may be more of an indictment of how incompletely current hypertension treatment algorithms target disease mechanisms.

## Barriers to adoption

The BP lowering capabilities of lorundrostat and baxdrostat compared with placebo will likely lead to their approval for the treatment of hypertension by global regulatory agencies—likely 2026 in the United States (US). However, such placebo comparisons are not enough to gain early widespread adoption among clinicians and, equally importantly, favorable coverage by payers in the US. As with most newly approved pharmacotherapies in the US, cost, prior authorization, and generic alternatives will present significant barriers to adoption.

The cost and prior authorization requirements of ASIs are not yet known, but the availability of generic alternatives is established. As noted above, mechanistically, ASIs should not have the adverse effect profile of a steroidal MRA, and theoretically, ASIs may also address the adverse non-genomic effects from aldosterone. However, whether these benefits truly translate to real-world outcomes, such as improved adherence or better cardiovascular outcomes, remains uncertain.

Likely the first consequence of increasing recognition of aldosterone dysregulation as a driver of BP elevations, and a push toward prescribing ASIs, is wider use of spironolactone, whether initiated by the prescriber or mandated by payers. Following regulatory approval for the treatment of hypertension in the US, we foresee initial use of ASIs limited to patients who are intolerant of spironolactone—approximately 10% in one cohort with resistant hypertension [11]. Cardiovascular outcome data, or BP-lowering efficacy in trials with active comparators, will be crucial to broader use of lorundrostat and baxdrostat.

A renewed interest by pharmaceutical companies in novel ways to treat hypertension is welcome. Beyond ASIs, multiple additional drugs to treat hypertension are under investigation including long-acting subcutaneously administered drugs that suppress hepatic angiotensinogen production (zilbesiran and tonlamarsen, among others), and those that agonize the glucagon-like peptide-1 receptor (orforglipron). Although many of these new therapies will lower BP, like ASIs, their widespread use is far from assured. They may ultimately gain traction due to benefits beyond BP reduction, such as assured patient adherence or other beneficial cardiometabolic effects, such as weight loss.

As hypertension care evolves from generic, one-size-fits-all pathways toward targeted, mechanism-based therapies, adoption hurdles remain. Yet the potential to improve BP control is considerable, and aldosterone synthase inhibition may be the catalyst that ushers in this transformation.

## Abbreviations:

ASI: aldosterone synthase inhibitor
BP: blood pressure
MRA: mineralocorticoid receptor antagonist
